# Supplementary material for: Bacteria can maintain rRNA operons solely on plasmids for hundreds of millions of years
Source: Nat Commun. 2023 Nov 14;14:7232. doi: 10.1038/s41467-023-42681-w (PMC10645730; doi:10.1038/s41467-023-42681-w)
Supplement: Supplementary file 1 — Supplementary Information [file 41467_2023_42681_MOESM1_ESM.pdf]

Supplementary Information

Bacteria can maintain rRNA operons solely on plasmids for hundreds of millions of years

Mizue Anda[1], Shun Yamanouchi[2], Salvatore Cosentino[1], Mitsuo Sakamoto[3], Moriya Ohkuma[3], Masako Takashima[3], Atsushi Toyoda[4], and Wataru Iwasaki[1,2,5-8]

*Corresponding authors:

Mizue Anda (anda@k.u-tokyo.ac.jp) or Wataru Iwasaki (iwasaki@k.u-tokyo.ac.jp)

This PDF file includes:

Supplementary Figures 1 to 9.

Supplementary Table 1.

Supplementary Figures

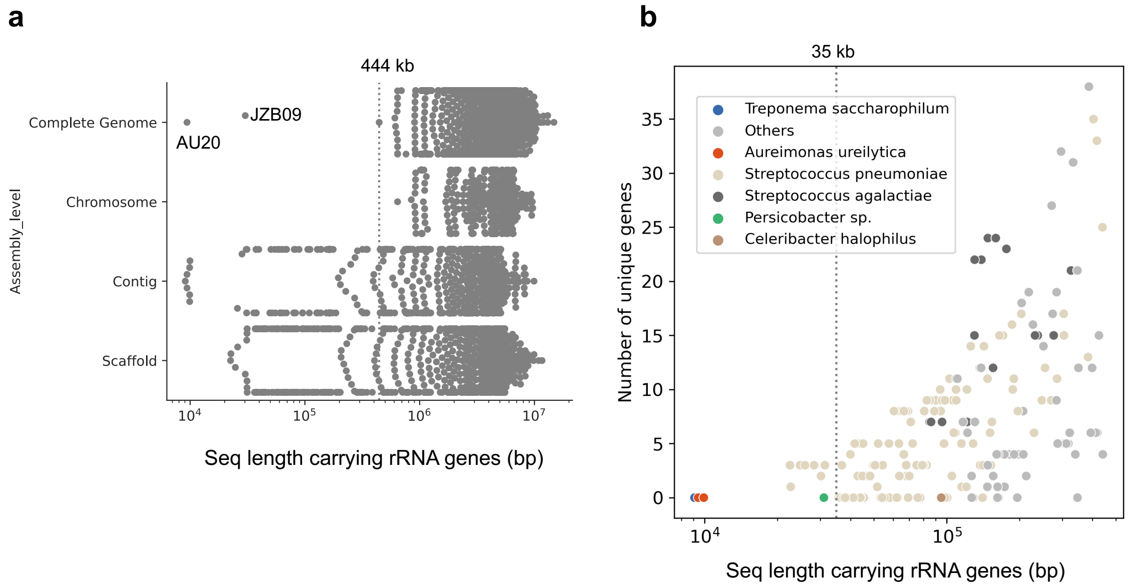

Supplementary Fig. 1. Contigs hit by the two selections of the genome database search. (a) 4,677 genomes whose annotated *rrn* operons were exclusively on contigs that encoded *rep* genes. This condition also hits sequences derived from chromosomes. (b) Of panel a, 298 contigs derived from contig/scaffold and less than 444 kb (minimal chromosome in panel a) were plotted. The number of unique genes was identified using GTDBtk identify. The chromosome-derived contigs increased as the sequence length increased (e.g., *Streptococcus peumoniae*, *Streptococcus agalactiae*, *Celeribacter halophilus*). Condition (contig < 35 kb and encoded no essential single-copy genes) contains only *Aureimonas*, *Treponema*, and *Persicobacter*.

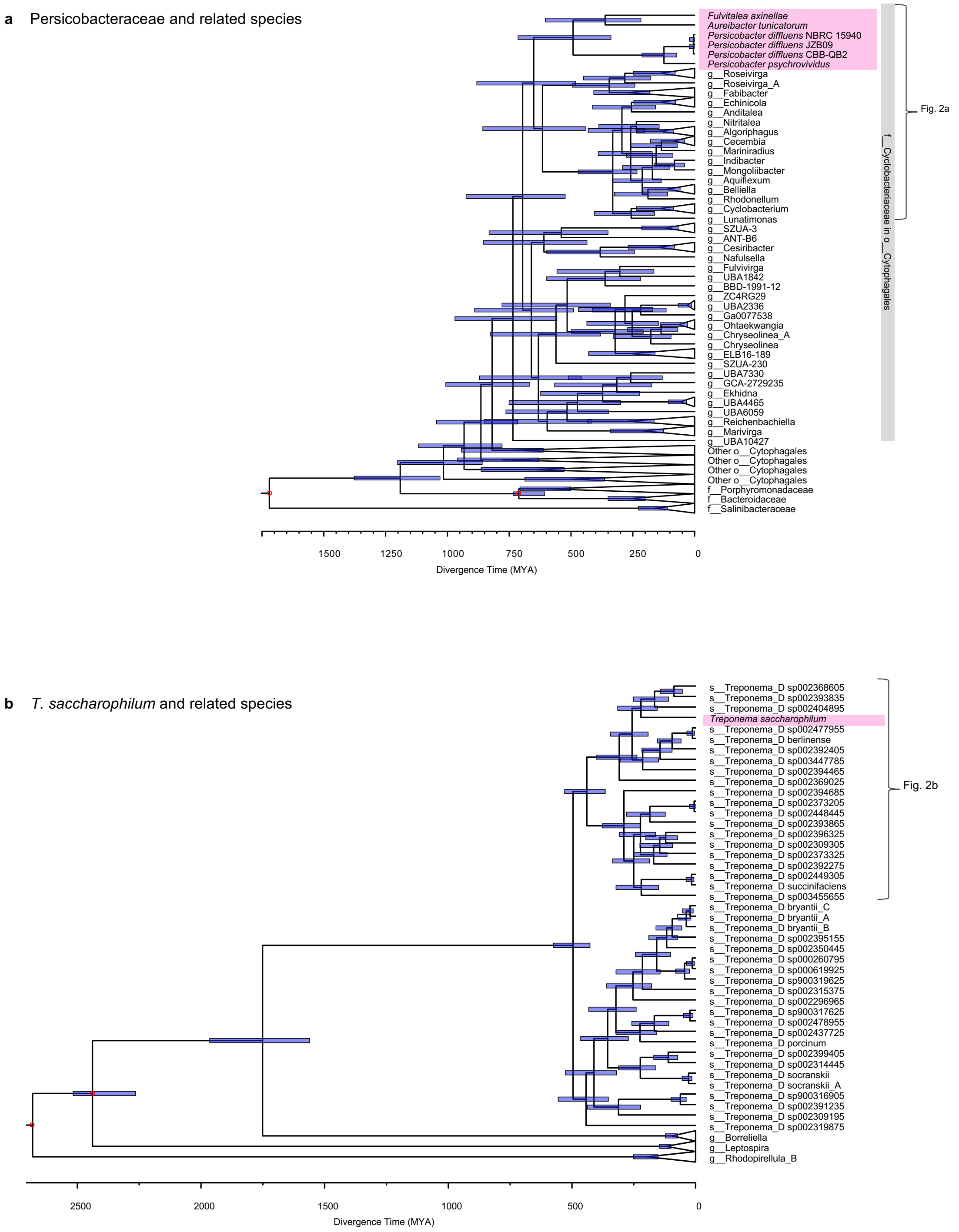
a Persicobacteraceae and related species
Fulvitalea axinellae
Aureibacter tunicatorum
Persicobacter diffluens NBRC 15940
Persicobacter diffluens JZB09
Persicobacter diffluens CBB-QB2
Persicobacter psychrovividus
g__Roseivirga
g__Roseivirga_A
g__Fabibacter
g__Echinicola
g__Anditalea
g__Nitritalea
g__Algoriphagus
g__Cecembia
g__Mariniradius
g__Indibacter
g__Mongoliibacter
g__Aquiflexum
g__Belliella
g__Rhodonellum
g__Cyclobacterium
g__Lunatimonas
g__SZUA-3
g__ANT-B6
g__Cesiribacter
g__Nafulsella
g__Fulvivirga
g__UBA1842
g__BBD-1991-12
g__ZC4RG29
g__UBA2336
g__Ga0077538
g__Ohtaekwangia
g__Chryseolinea_A
g__Chryseolinea
g__ELB16-189
g__SZUA-230
g__UBA7330
g__GCA-2729235
g__Ekhidna
g__UBA4465
g__UBA6059
g__Reichenbachiella
g__Marivirga
g__UBA10427
Other o__Cytophagales
Other o__Cytophagales
Other o__Cytophagales
Other o__Cytophagales
f__Porphyromonadaceae
f__Bacteroidaceae
f__Salinibacteraceae
f__Cyclobacteriaceae in o__Cytophagales
Fig. 2a
1500
1250
1000
750
500
250
0
Divergence Time (MYA)
b T. saccharophilum and related species
s__Treponema_D sp002368605
s__Treponema_D sp002393835
s__Treponema_D sp002404895
Treponema saccharophilum
s__Treponema_D sp002477955
s__Treponema_D berlinense
s__Treponema_D sp002392405
s__Treponema_D sp003447785
s__Treponema_D sp002394465
s__Treponema_D sp002369025
s__Treponema_D sp002394685
s__Treponema_D sp002373205
s__Treponema_D sp002448445
s__Treponema_D sp002393865
s__Treponema_D sp002396325
s__Treponema_D sp002309305
s__Treponema_D sp002373325
s__Treponema_D sp002392275
s__Treponema_D sp002449305
s__Treponema_D succinifaciens
s__Treponema_D sp003455655
s__Treponema_D bryantii_C
s__Treponema_D bryantii_A
s__Treponema_D bryantii_B
s__Treponema_D sp002395155
s__Treponema_D sp002350445
s__Treponema_D sp000260795
s__Treponema_D sp000619925
s__Treponema_D sp900319625
s__Treponema_D sp002315375
s__Treponema_D sp002296965
s__Treponema_D sp900317625
s__Treponema_D sp002478955
s__Treponema_D sp002437725
s__Treponema_D porcinum
s__Treponema_D sp002399405
s__Treponema_D sp002314445
s__Treponema_D socranskii
s__Treponema_D socranskii_A
s__Treponema_D sp900316905
s__Treponema_D sp002391235
s__Treponema_D sp002309195
s__Treponema_D sp002319875
g__Borreliella
g__Leptospira
g__Rhodopirellula_B
Fig. 2b
2500
2000
1500
1000
500
0
Divergence Time (MYA)

c *A. ureilytica* and related species

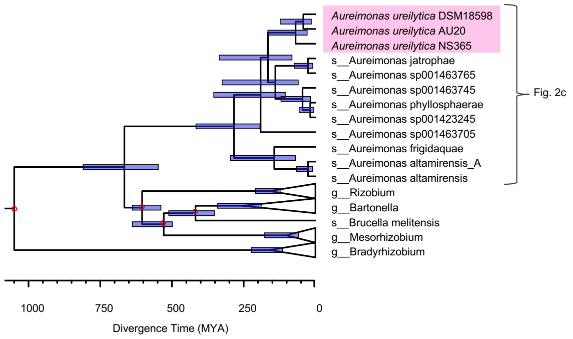

d *O. saccharovorans* and related species

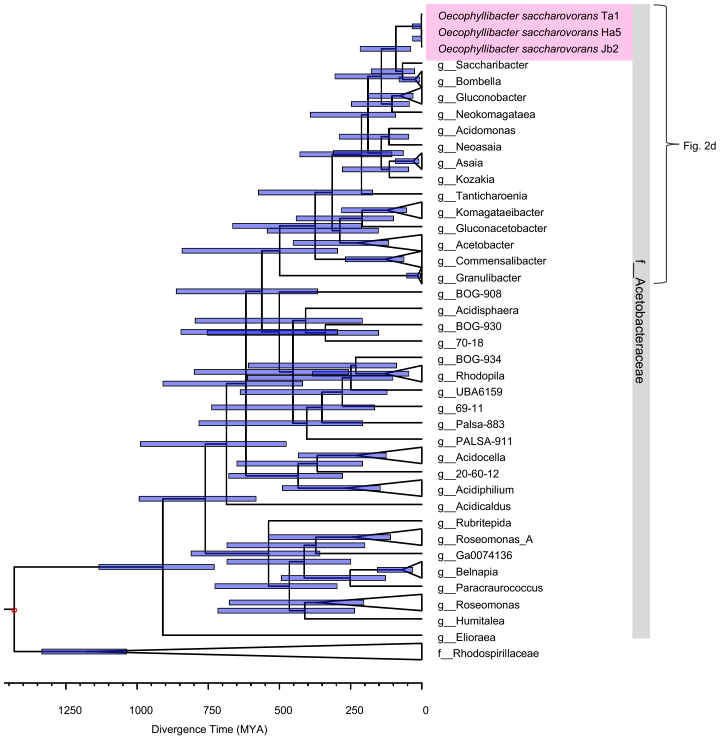

Supplementary Fig. 2. Original phylogenetic trees in Fig. 2. (a) Persicobacteraceae species, (b) *T. saccharophilum*, (c) *A. ureilytica*, and (d) *O. saccharovorans.* Pink indicates bacteria without chromosomal *rrn* operons. Blue bars show confidence intervals of RelTime estimates which contain the actual time with 94% probability[85]. Species and genus names follow GTDB taxonomy, but names of bacteria without chromosomal *rrn* operons follow IJSEM for consistency with the main text. Red open circles on nodes are calibration points. Genomes used for divergence time estimation are shown in Supplementary Data 4.

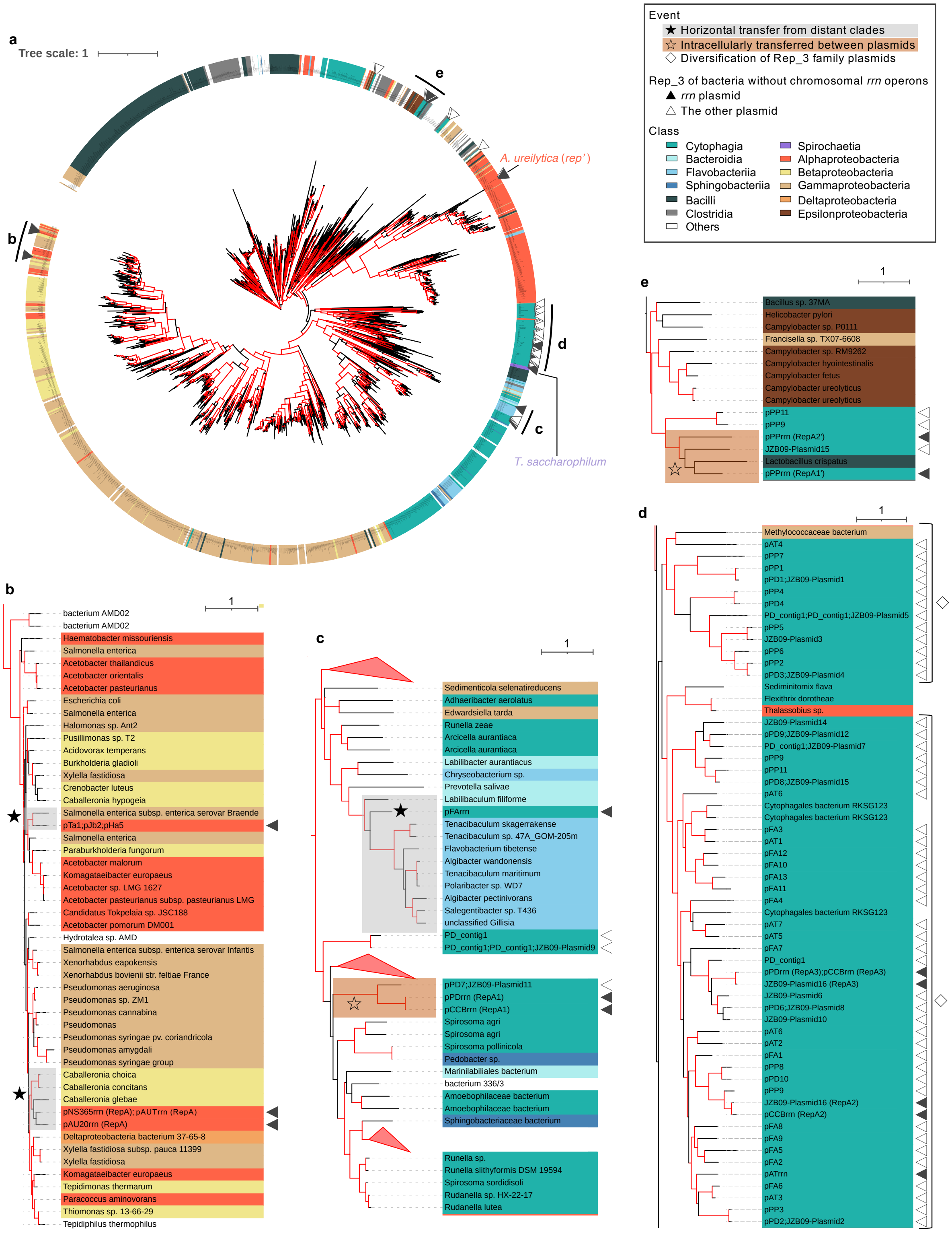

Supplementary Fig. 3. Phylogenetic trees of representative Rep_3-family genes in bacteria without chromosomal *rrn* operons. Closed and open triangles indicate Rep_3 genes on *rrn*

plasmids and other replicons of bacteria without chromosomal *rrn* operons, respectively. Colors represent phylogenetic classes of host bacteria. Red branches represent bootstrap values > 70. See Fig. 1a-e for gene names. Tip labels show replicon names of bacteria without chromosomal *rrn* operons or representative sequences of clusters.

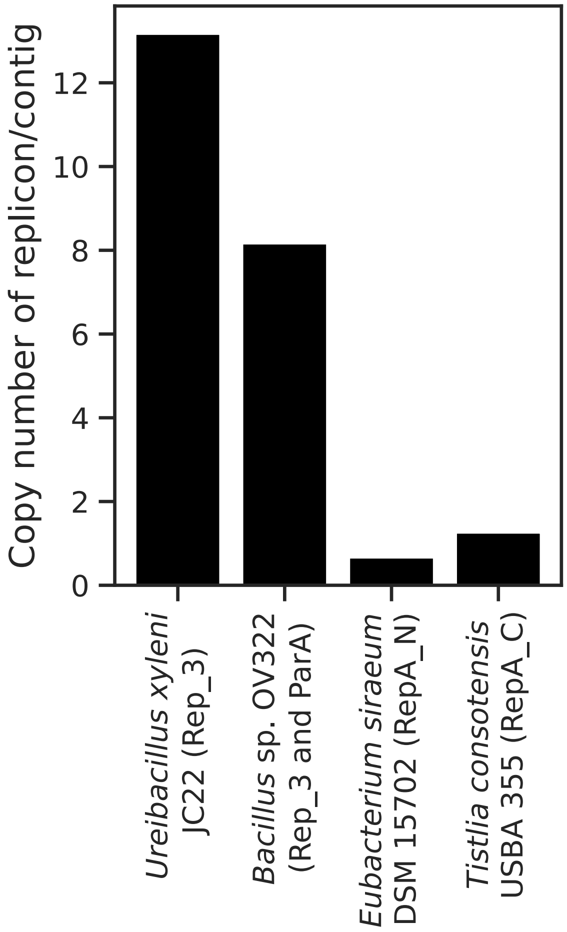

Supplementary Fig. 4. Estimated relative copy numbers of plasmids that encode Rep_3-family or other *rep* genes in bacteria with chromosomal *rrn* operons.

Persicobacteraceae family and related species

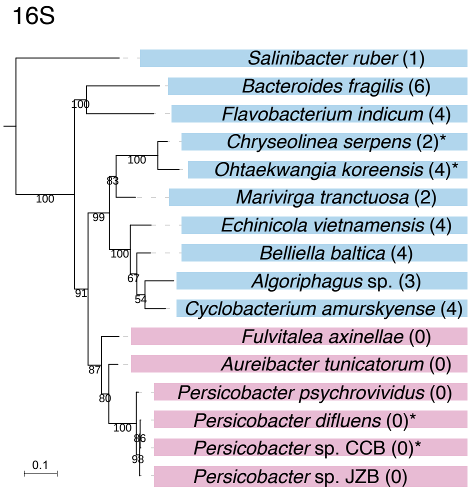

Supplementary Fig. 5. Phylogenetic tree of 16S rRNA genes of Persicobacteraceae species and their related species. Pink indicates bacteria without chromosomal *rrn* operons. Light blue indicates bacteria with chromosomal *rrn* operons. Numbers in parentheses are numbers of chromosomal *rrn* operons. Asterisks indicate genomes whose assembly levels are scaffold. Bootstrap values of >50% are shown. Scale bars indicate substitution numbers per site. See Supplementary Data 1 for strain names.

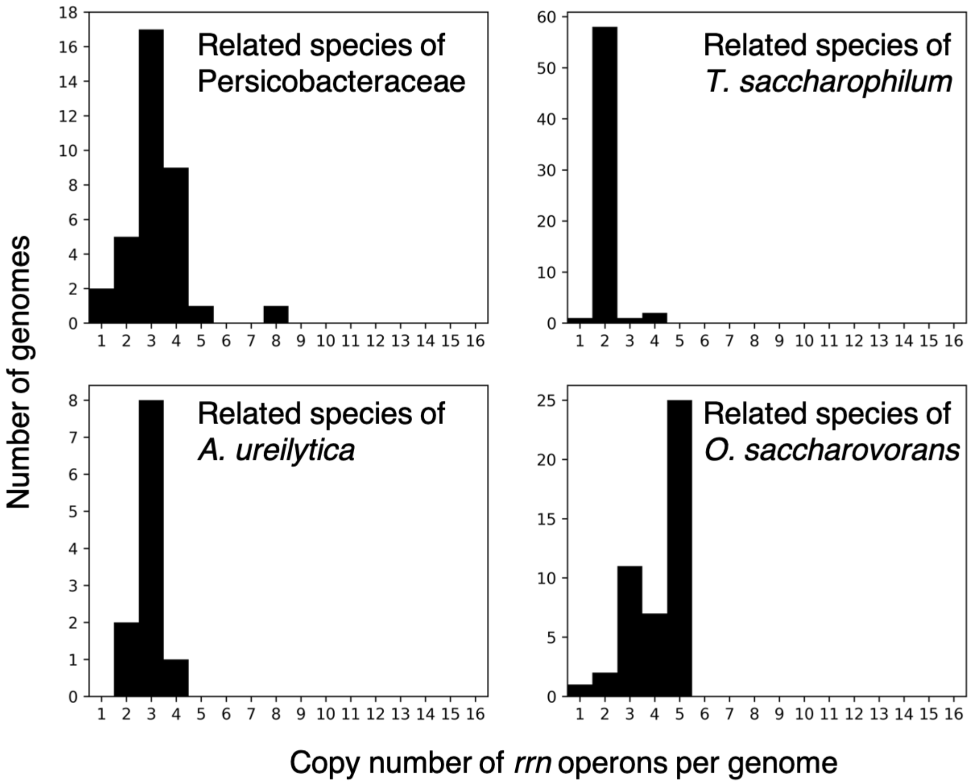

Supplementary Fig. 6. Copy numbers of *rrn* operons in related species of bacteria without chromosomal *rrn* operons in the four clades. Complete genomes were used for counting, except for the related species of *A. ureilytica* (Aurantimonadaceae), whose copy numbers were estimated by Southern hybridization and draft genomes[11]. Note that a related strain of Persicobacteraceae (*Flammeovirga* sp. MY04) with 8 copies of *rrn* operons is not in a sister clade of Persicobacteraceae and not included in the phylogenetic tree of Supplementary Fig. 8a.

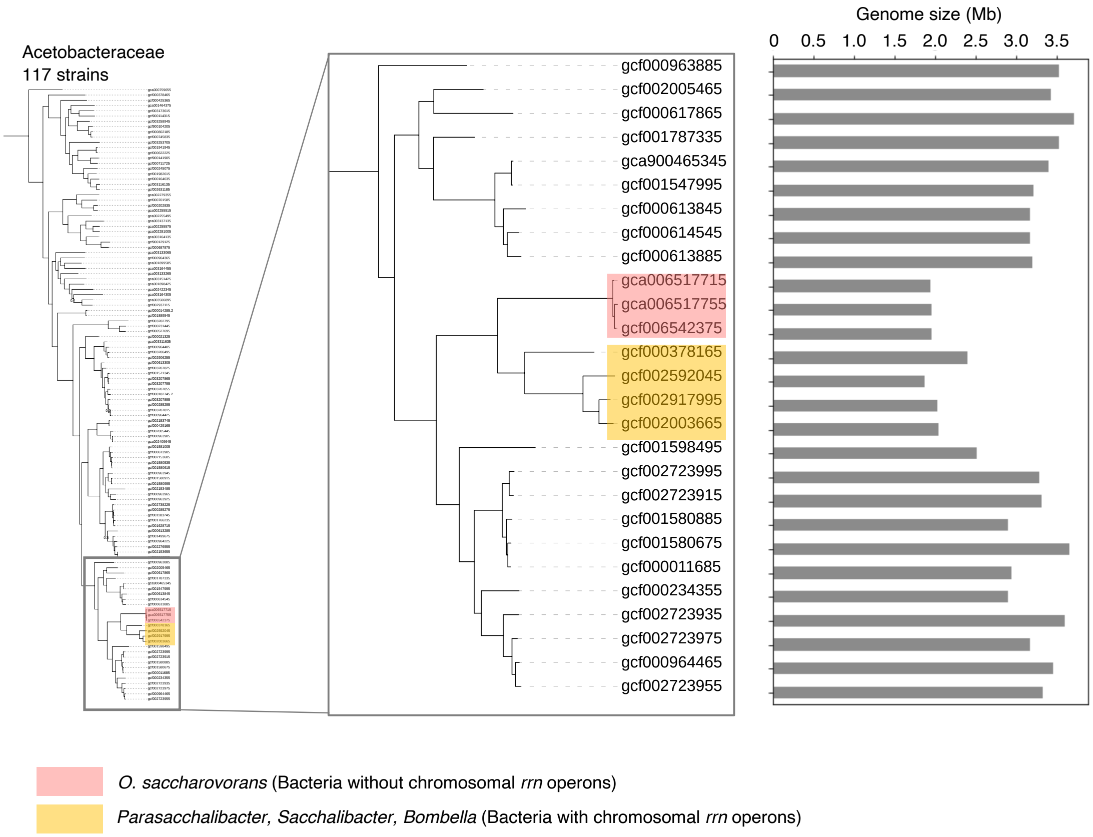

Supplementary Fig. 7. Genome reduction of *Oecophyllibacter saccharovorans* and related species. Phylogenetic tree and genome sizes of *O. saccharovorans* (bacteria without chromosomal *rrn* operon, pink) and related species *Parasacchalibacter, Sacchalibacter*, and *Bombella* (bacteria with chromosomal *rrn* operons, orange) are shown.

a Persicobacteraceae and related species

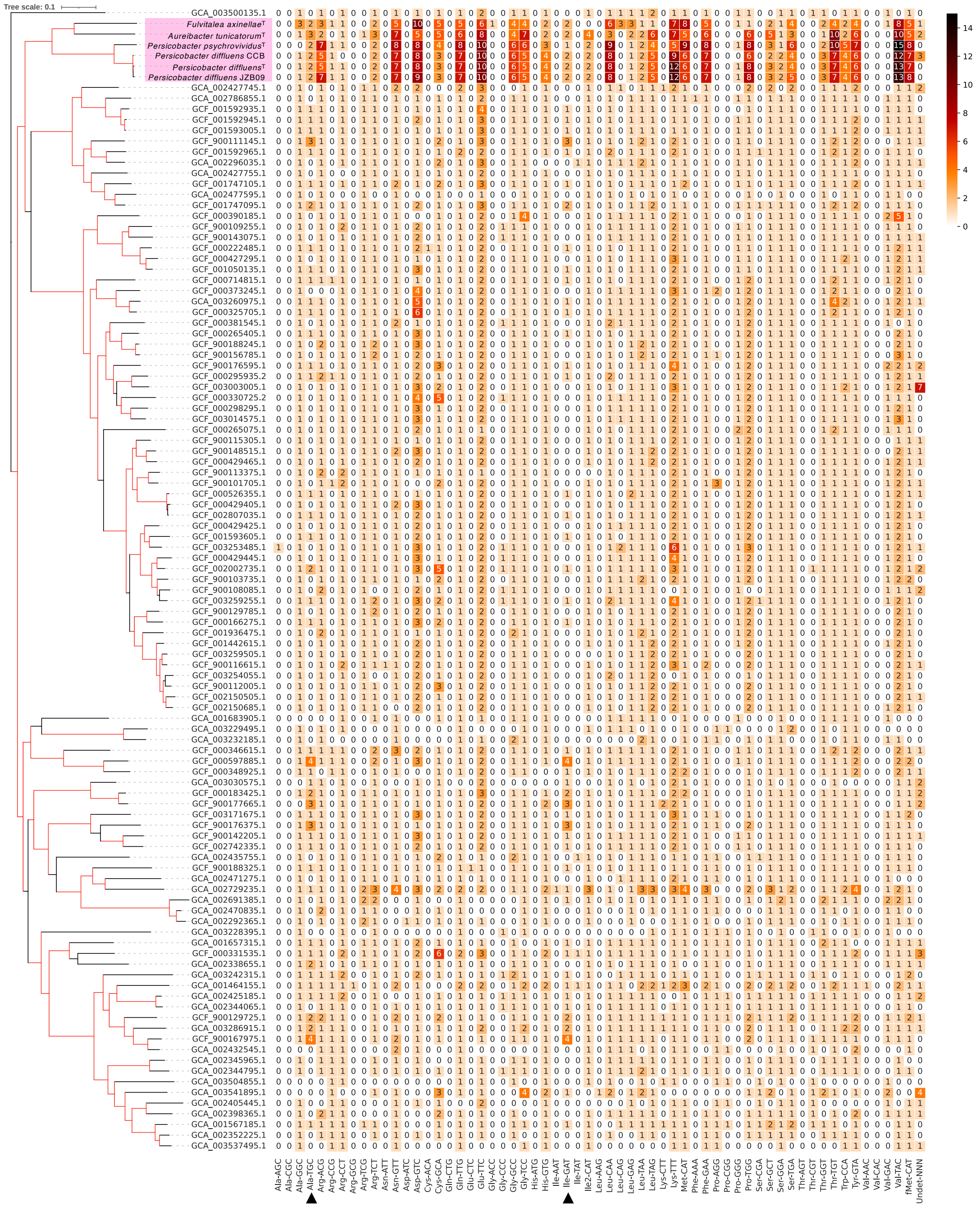

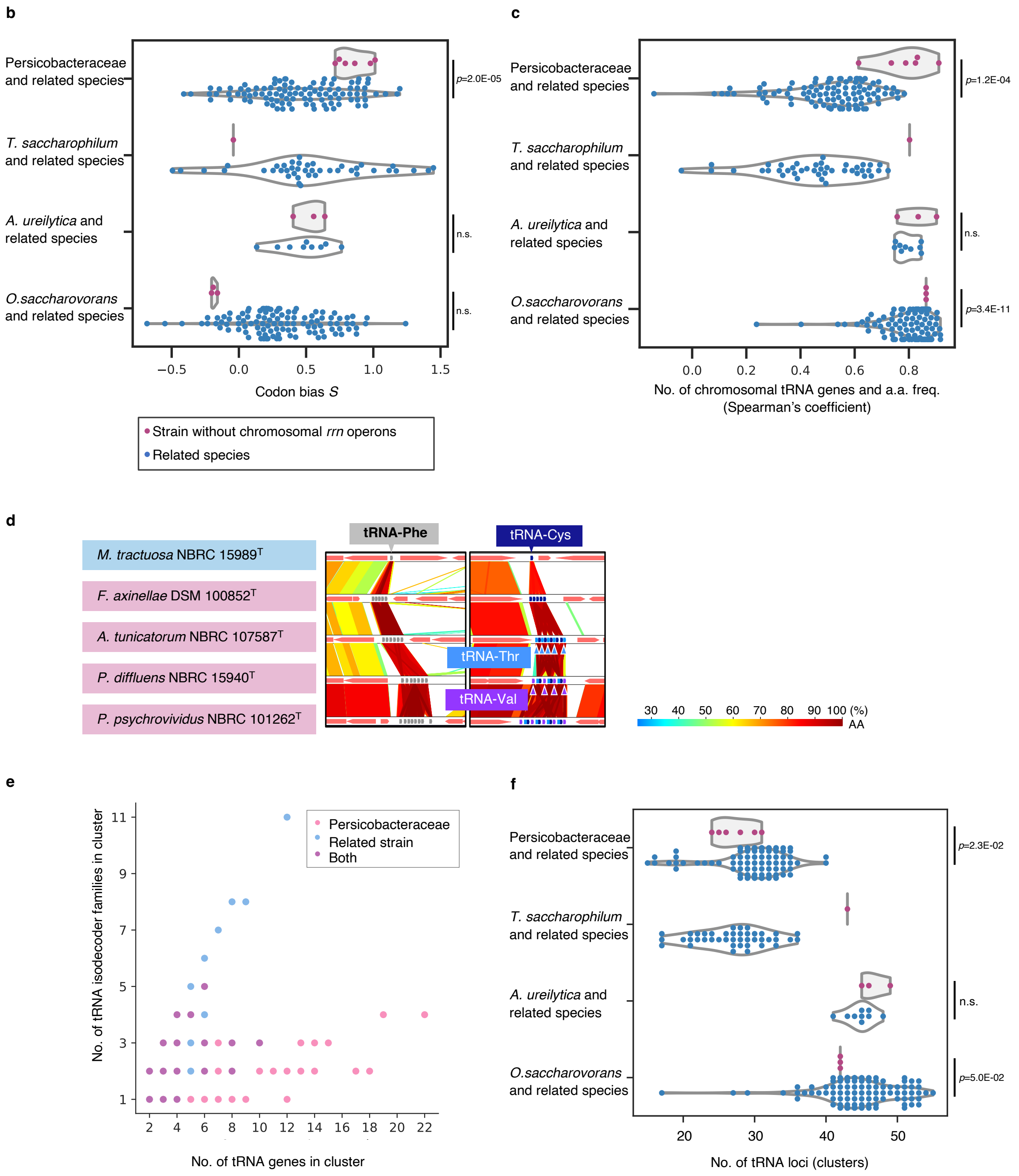

Supplementary Fig. 8. tRNA genes in bacteria without chromosomal *rrn* plasmids. (a) Gene numbers per anticodon in Persicobacteraceae and related species. Phylogenetic tree was inferred by GTDBtk. Filled triangles show tRNA genes inserted into *rrn* operons in Persicobacteraceae. (b) Codon bias *S* in four independently evolved bacterial clades and related species. (c) Spearman's rank correlations between the amino acid frequency of all

protein-coding genes and copy numbers of chromosomal tRNA genes. (d) Examples of tandem repeats of chromosomal tRNA genes in tRNA clusters in Persicobacteraceae species. (e) Numbers of tRNA isodecoder families and genes in tRNA clusters in Persicobacteraceae species. (f) Numbers of tRNA loci (gene clusters) in four independently evolved bacterial clades and related species. (b, c, f) Comparison with related species for the four independently evolved bacterial clades: Persicobacteraceae (n=6) and related species (n=101), *T. saccharophirum* (n=1) and related species (n=43), *A. ureilytica* (n=3) and related species (n=18), and *O. saccharovorans* (n=3) and related species (n=126). P value was calculated using Mann-Whitney U-test (one sided). n.s.: Not significant (p>0.05). Source data are provided as a Source Data file.

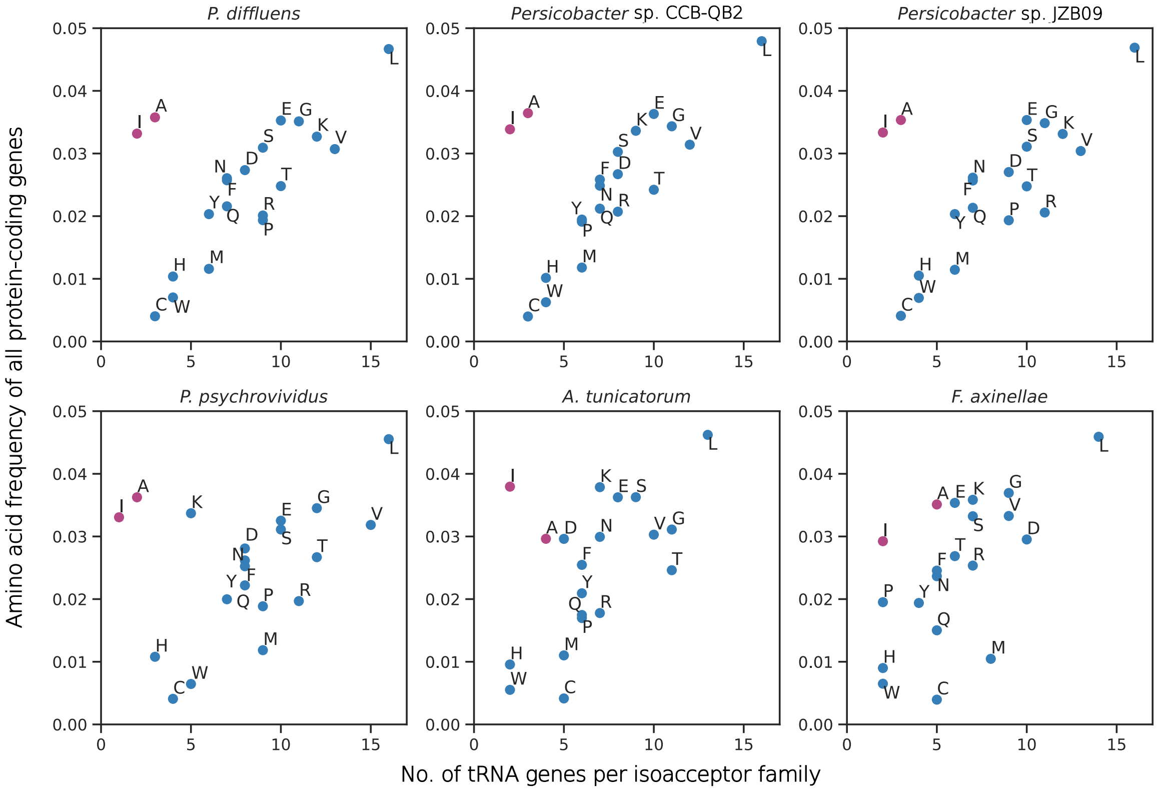

Supplementary Fig. 9. Scatter plot of tRNA gene copy number and amino-acid composition of all protein-coding genes in Persicobacteraceae.

Supplementary Table

Supplementary Table 1. Genome sequencing statistics per replicon.

Strain	Replicon or contig	Size (bp)	GC (%)	CDS	rRNA	tRNA	tmRNA	Repeat	Topology	Accession no.	Description
Persicobacter diffluens NBRC 15940(T)	Chromosome	3,629,155	42	2,826	0	155	0	0	Circular	BQKE01000001	
	pPD1	1,518,630	42	915	0	0	0	0	Circular	BQKE01000002	
	pPD2	803,428	41	560	0	6	0	0	Circular	BQKE01000003	
	pPD3	470,181	42	249	0	0	0	0	Circular	BQKE01000004	
	pPD4	411,990	42	205	0	0	0	0	Circular	BQKE01000005	
	Contig1	284,208	40	260	0	0	0	0	Linear	BQKE01000006	
	pPD6	97,057	39	85	0	0	0	0	Circular	BQKE01000007	
	pPD7	55,670	40	56	0	0	0	0	Circular	BQKE01000008	
	pPD8	49,222	42	48	0	0	0	0	Circular	BQKE01000009	
	pPD9	42,118	41	36	0	0	0	0	Circular	BQKE01000010	
	pPD10	40,323	41	23	0	0	0	0	Circular	BQKE01000011	
	pPD*rrn*	29,644	47	11	9	4	0	0	Circular	BQKE01000012	*rrn* plasmid
	Contig2	27,938	38	26	0	0	0	0	Linear	BQKE01000013	
	Contig3	27,204	40	22	0	0	0	0	Linear	BQKE01000014	
	Contig4	17,556	40	22	0	0	0	0	Linear	BQKE01000015	
	Contig5	8,373	35	10	0	0	0	0	Linear	BQKE01000016	
	Total	7,512,697		5,354	9	165	0	0			
Persicobacter psychrovividus NBRC 101262(T)	Chromosome	3,216,249	43	2,590	0	166	0	0	Circular	AP025292	
	pPP1	1,068,661	44	687	0	0	0	0	Circular	AP025293	
	pPP2	404,592	40	289	0	0	0	0	Circular	AP025294	
	pPP3	379,741	42	321	0	5	0	0	Circular	AP025295	
	pPP4	234,976	44	115	0	0	0	0	Circular	AP025296	
	pPP5	206,276	42	113	0	0	0	0	Circular	AP025297	
	pPP6	157,247	42	119	0	0	0	1	Circular	AP025298	
	pPP7	145,245	44	77	0	0	0	0	Circular	AP025299	
	pPP8	143,728	39	89	0	0	0	0	Circular	AP025300	
	pPP9	66,375	40	60	0	0	0	0	Circular	AP025301	
	pPP10	53,207	41	51	0	0	0	0	Circular	AP025302	
	pPP11	46,459	39	29	0	0	0	3	Circular	AP025303	
	pPP*rrn*	25,858	47	11	6	2	0	0	Circular	AP025304	*rrn* plasmid
	Total	6,148,614		4,551	6	173	0	4			
Aureibacter tunicatorum NBRC 107587(T)	Chromosome	4,795,222	37	3,843	0	137	0	0	Circular	AP025305	
	pAT1	615,057	36	518	0	0	0	0	Circular	AP025306	
	pAT2	295,322	37	233	0	0	0	0	Circular	AP025307	
	pAT3	212,319	36	125	0	0	0	0	Circular	AP025308	
	pAT4	104,573	37	68	0	0	0	0	Circular	AP025309	
	pAT5	61,258	35	56	0	0	0	0	Circular	AP025310	
	pAT6	58,368	36	51	0	0	0	2	Circular	AP025311	
	pAT7	15,402	34	15	0	0	0	0	Circular	AP025312	
	pAT*rrn*	13,754	45	1	6	4	0	1	Circular	AP025313	*rrn* plasmid
	Total	6,171,275		4,910	6	141	0	3			
Fulvitalea axinellae DSM 100852(T)	Chromosome	4,866,544	47	3,686	0	119	0	0	Circular	AP025314	
	pFA1	554,072	45	385	0	0	0	0	Circular	AP025315	
	pFA2	437,430	47	246	0	3	0	1	Circular	AP025316	
	pFA3	324,713	45	222	0	0	0	1	Circular	AP025317	
	pFA4	309,146	46	211	0	0	0	0	Circular	AP025318	
	pFA5	249,895	46	179	0	0	0	0	Circular	AP025319	
	pFA6	226,742	46	154	0	0	0	0	Circular	AP025320	
	pFA7	107,153	49	49	0	0	0	0	Circular	AP025321	
	pFA8	64,707	46	59	0	0	0	2	Circular	AP025322	
	pFA9	62,265	47	55	0	0	0	0	Circular	AP025323	
	pFA10	54,475	47	40	0	0	0	0	Circular	AP025324	
	pFA11	45,438	44	39	0	0	0	0	Circular	AP025325	
	pFA12	43,902	47	34	0	0	0	0	Circular	AP025326	
	pFA13	37,745	50	43	0	0	0	0	Circular	AP025327	
	pFA*rrn*	13,698	51	1	6	4	0	0	Circular	AP025328	*rrn* plasmid
	Total	7,397,925		5,403	6	126	0	4			
Marivirga tractuosa NBRC 15989(T)	Chromosome	4,530,163	36	3,724	6	39	0	0	Circular	AP025329	
	pMT1	4,916	40	8	0	0	0	0	Circular	AP025330	
	Total	4,535,079		3,732	6	39	0	0			
Treponema bryantii JCM 32280(T)	Chromosome	3,431,497	38	2,923	12	41	0	0	Circular	AP025286	
	pTB1	85,103	32	83	0	0	0	0	Circular	AP025287	
	Total	3,516,600		3,006	12	41	0	0			
Treponema saccharophilum JCM 32279(T)	Chromosome	2,645,182	54	2,159	0	56	0	4	Circular	AP025288	
	pTS1	423,813	48	397	0	2	0	0	Circular	AP025289	
	pTS2	381,873	51	344	0	1	0	1	Circular	AP025290	
	pTS*rrn*	8,452	54	3	3	1	0	0	Circular	AP025291	*rrn* plasmid
	Total	3,459,320		2,903	3	60	0	5			
