# Supplementary material for: Bacteria can maintain rRNA operons solely on plasmids for hundreds of millions of years
Source: Nat Commun. 2023 Nov 14;14:7232. doi: 10.1038/s41467-023-42681-w (PMC10645730; doi:10.1038/s41467-023-42681-w)
Supplement: Supplementary file 3 — Description of Additional Supplementary Files [file 41467_2023_42681_MOESM3_ESM.pdf]

Description of Additional Supplementary Files:

Supplementary Data 1: Strains used for phylogenetic analysis in Fig. 1fg.

Supplementary Data 2: Conserved single-copy genes extracted by bcgtree.

Supplementary Data 3: Tree topology tests for phylogenetic trees in Fig. 1fg.

Supplementary Data 4: Genomes used for divergence time estimation in Fig. 2a–d and Supplementary Fig. 2.

Supplementary Data 5: Seventeen Pfam families whose primary function is plasmidial replication.

Supplementary Data 6: Sequences used for phylogenetic analysis of Repfamily genes

Supplementary Data 7: Pfam families detected from 156 Rep sequences[20].

Supplementary Data 8: Sequences, alignment, and tree files for the phylogenetic tree in Supplementary Fig. 3.
